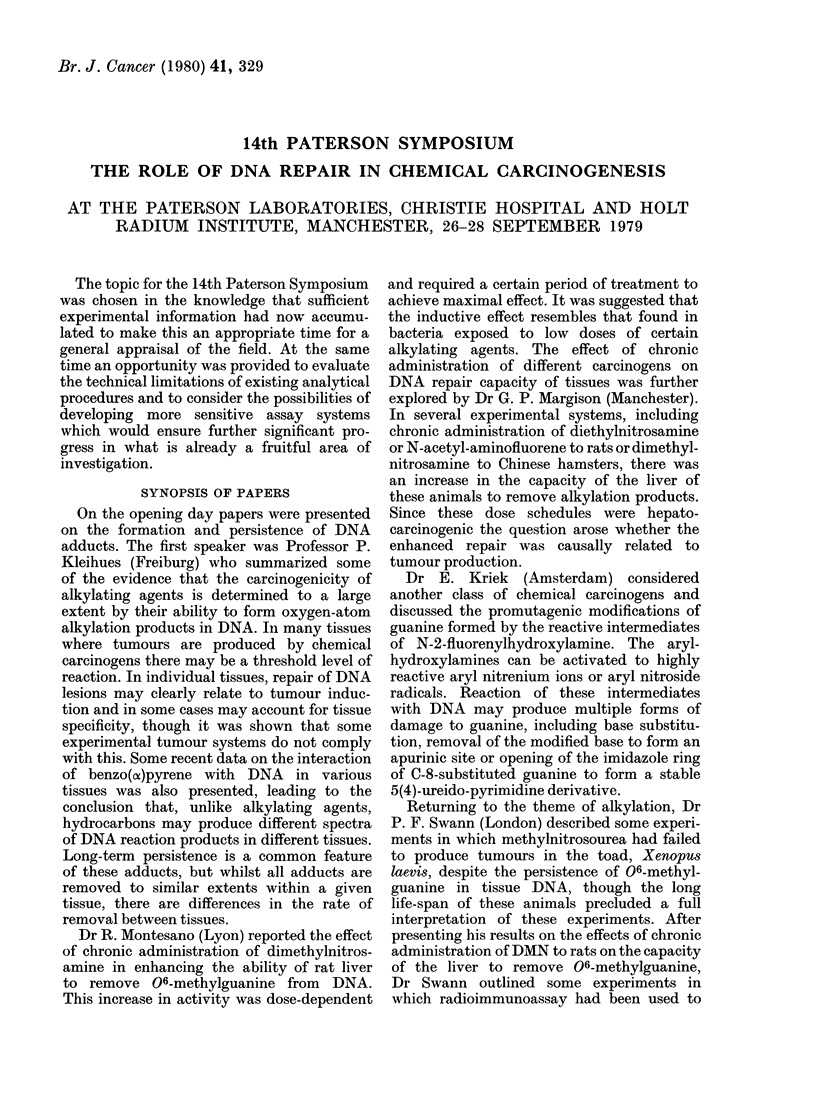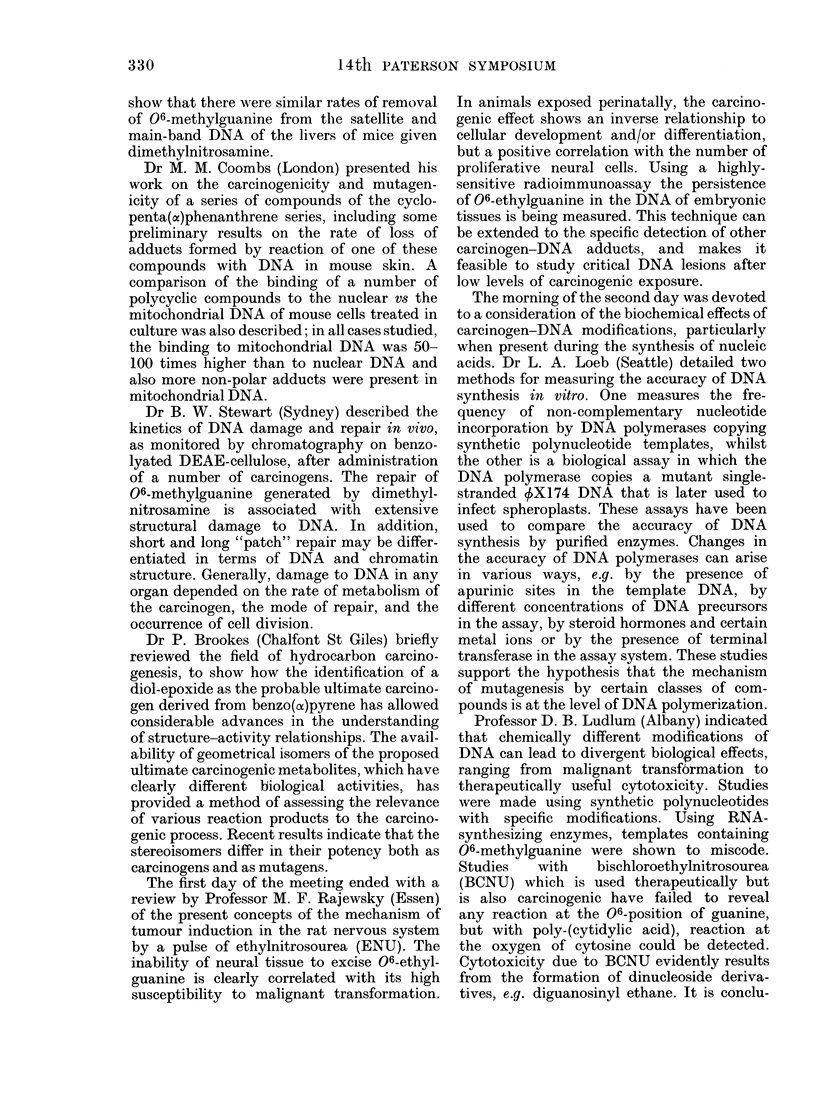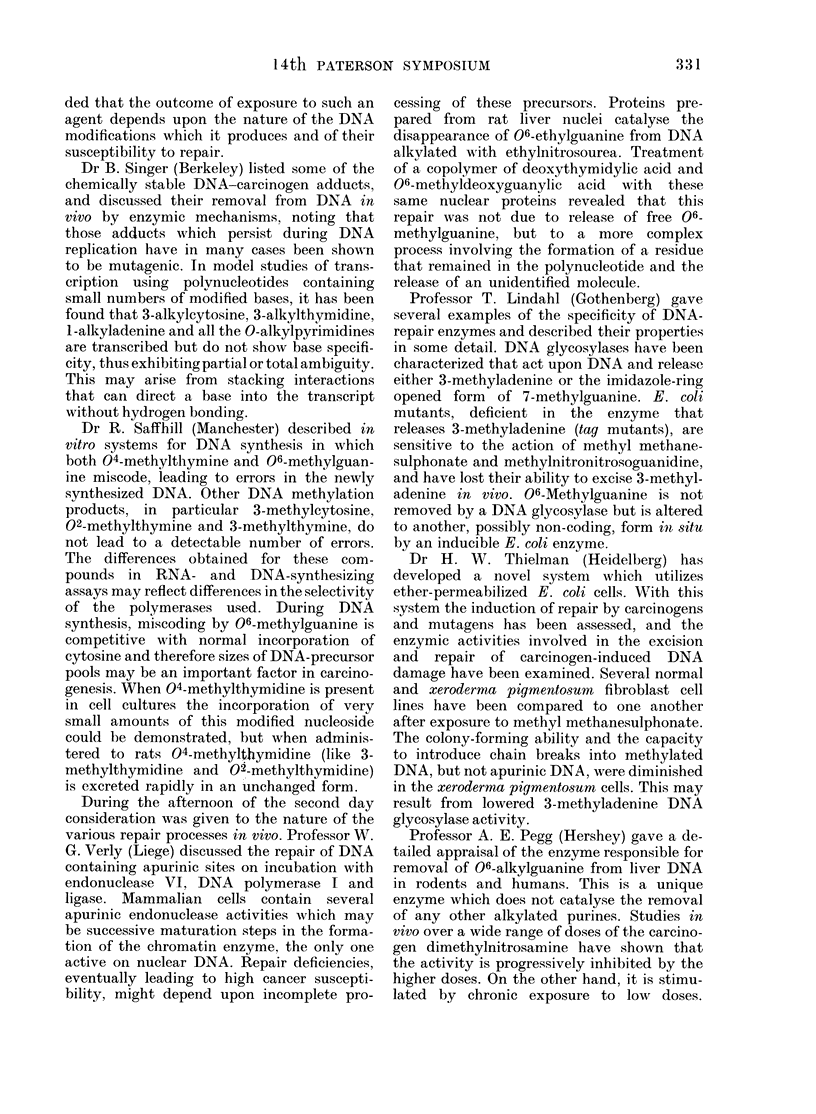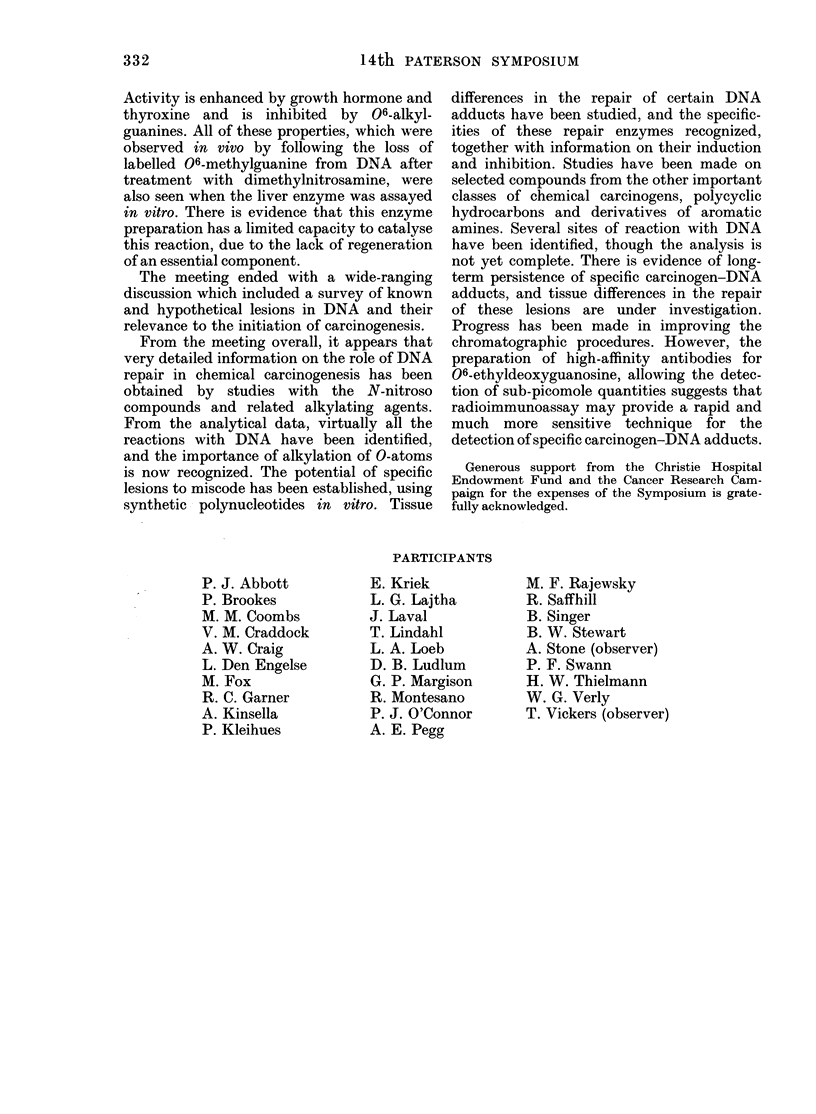# Proceedings of 14th Paterson Symposium The role of DNA repair in carcinogenesis

**Published:** 1980-02

**Authors:** 


*Br. J. Cancer* (1980) **41**, 329

### 14th PATERSON SYMPOSIUM

### THE ROLE OF DNA REPAIR IN CHEMICAL CARCINOGENESIS

### AT THE PATERSON LABORATORIES, CHRISTIE HOSPITAL AND HOLT RADIUM INSTITUTE, MANCHESTER, 26–28 SEPTEMBER 1979

The topic for the 14th Paterson Symposium was chosen in the knowledge that sufficient experimental information had now accumulated to make this an appropriate time for a general appraisal of the field. At the same time an opportunity was provided to evaluate the technical limitations of existing analytical procedures and to consider the possibilities of developing more sensitive assay systems which would ensure further significant progress in what is already a fruitful area of investigation.

### SYNOPSIS OF PAPERS

On the opening day papers were presented on the formation and persistence of DNA adducts. The first speaker was Professor P. Kleihues (Freiburg) who summarized some of the evidence that the carcinogenicity of alkylating agents is determined to a large extent by their ability to form oxygen-atom alkylation products in DNA. In many tissues where tumours are produced by chemical carcinogens there may be a threshold level of reaction. In individual tissues, repair of DNA lesions may clearly relate to tumour induction and in some cases may account for tissue specificity, though it was shown that some experimental tumour systems do not comply with this. Some recent data on the interaction of benzo(α)pyrene with DNA in various tissues was also presented, leading to the conclusion that, unlike alkylating agents, hydrocarbons may produce different spectra of DNA reaction products in different tissues. Long-term persistence is a common feature of these adducts, but whilst all adducts are removed to similar extents within a given tissue, there are differences in the rate of removal between tissues.

Dr R. Montesano (Lyon) reported the effect of chronic administration of dimethylnitrosamine in enhancing the ability of rat liver to remove $O^6$-methylguanine from DNA. This increase in activity was dose-dependent and required a certain period of treatment to achieve maximal effect. It was suggested that the inductive effect resembles that found in bacteria exposed to low doses of certain alkylating agents. The effect of chronic administration of different carcinogens on DNA repair capacity of tissues was further explored by Dr G. P. Margison (Manchester). In several experimental systems, including chronic administration of diethylnitrosamine or N-acetyl-aminofluorene to rats or dimethylnitrosamine to Chinese hamsters, there was an increase in the capacity of the liver of these animals to remove alkylation products. Since these dose schedules were hepatocarcinogenic the question arose whether the enhanced repair was causally related to tumour production.

Dr E. Kriek (Amsterdam) considered another class of chemical carcinogens and discussed the promutagenic modifications of guanine formed by the reactive intermediates of N-2-fluorenylhydroxylamine. The arylhydroxylamines can be activated to highly reactive aryl nitrenium ions or aryl nitroside radicals. Reaction of these intermediates with DNA may produce multiple forms of damage to guanine, including base substitution, removal of the modified base to form an apurinic site or opening of the imidazole ring of C-8-substituted guanine to form a stable 5(4)-ureido-pyrimidine derivative.

Returning to the theme of alkylation, Dr P. F. Swann (London) described some experiments in which methylnitrosourea had failed to produce tumours in the toad, *Xenopus laevis*, despite the persistence of $O^6$-methylguanine in tissue DNA, though the long life-span of these animals precluded a full interpretation of these experiments. After presenting his results on the effects of chronic administration of DMN to rats on the capacity of the liver to remove $O^6$-methylguanine, Dr Swann outlined some experiments in which radioimmunoassay had been used to show that there were similar rates of removal of $O^6$-methylguanine from the satellite and main-band DNA of the livers of mice given dimethylnitrosamine.

Dr M. M. Coombs (London) presented his work on the carcinogenicity and mutagenicity of a series of compounds of the cyclopenta(α)phenanthrene series, including some preliminary results on the rate of loss of adducts formed by reaction of one of these compounds with DNA in mouse skin. A comparison of the binding of a number of polycyclic compounds to the nuclear *vs* the mitochondrial DNA of mouse cells treated in culture was also described; in all cases studied, the binding to mitochondrial DNA was 50–100 times higher than to nuclear DNA and also more non-polar adducts were present in mitochondrial DNA.

Dr B. W. Stewart (Sydney) described the kinetics of DNA damage and repair *in vivo*, as monitored by chromatography on benzolyated DEAE-cellulose, after administration of a number of carcinogens. The repair of $O^6$-methylguanine generated by dimethylnitrosamine is associated with extensive structural damage to DNA. In addition, short and long "patch" repair may be differentiated in terms of DNA and chromatin structure. Generally, damage to DNA in any organ depended on the rate of metabolism of the carcinogen, the mode of repair, and the occurrence of cell division.

Dr P. Brookes (Chalfont St Giles) briefly reviewed the field of hydrocarbon carcinogenesis, to show how the identification of a diol-epoxide as the probable ultimate carcinogen derived from benzo(α)pyrene has allowed considerable advances in the understanding of structure–activity relationships. The availability of geometrical isomers of the proposed ultimate carcinogenic metabolites, which have clearly different biological activities, has provided a method of assessing the relevance of various reaction products to the carcinogenic process. Recent results indicate that the stereoisomers differ in their potency both as carcinogens and as mutagens.

The first day of the meeting ended with a review by Professor M. F. Rajewsky (Essen) of the present concepts of the mechanism of tumour induction in the rat nervous system by a pulse of ethylnitrosourea (ENU). The inability of neural tissue to excise $O^6$-ethylguanine is clearly correlated with its high susceptibility to malignant transformation. In animals exposed perinatally, the carcinogenic effect shows an inverse relationship to cellular development and/or differentiation, but a positive correlation with the number of proliferative neural cells. Using a highly-sensitive radioimmunoassay the persistence of $O^6$-ethylguanine in the DNA of embryonic tissues is being measured. This technique can be extended to the specific detection of other carcinogen–DNA adducts, and makes it feasible to study critical DNA lesions after low levels of carcinogenic exposure.

The morning of the second day was devoted to a consideration of the biochemical effects of carcinogen–DNA modifications, particularly when present during the synthesis of nucleic acids. Dr L. A. Loeb (Seattle) detailed two methods for measuring the accuracy of DNA synthesis *in vitro*. One measures the frequency of non-complementary nucleotide incorporation by DNA polymerases copying synthetic polynucleotide templates, whilst the other is a biological assay in which the DNA polymerase copies a mutant single-stranded $\phi$X174 DNA that is later used to infect spheroplasts. These assays have been used to compare the accuracy of DNA synthesis by purified enzymes. Changes in the accuracy of DNA polymerases can arise in various ways, *e.g.* by the presence of apurinic sites in the template DNA, by different concentrations of DNA precursors in the assay, by steroid hormones and certain metal ions or by the presence of terminal transferase in the assay system. These studies support the hypothesis that the mechanism of mutagenesis by certain classes of compounds is at the level of DNA polymerization.

Professor D. B. Ludlum (Albany) indicated that chemically different modifications of DNA can lead to divergent biological effects, ranging from malignant transformation to therapeutically useful cytotoxicity. Studies were made using synthetic polynucleotides with specific modifications. Using RNA-synthesizing enzymes, templates containing $O^6$-methylguanine were shown to miscode. Studies with bischloroethylnitrosourea (BCNU) which is used therapeutically but is also carcinogenic have failed to reveal any reaction at the $O^6$-position of guanine, but with poly-(cytidylic acid), reaction at the oxygen of cytosine could be detected. Cytotoxicity due to BCNU evidently results from the formation of dinucleoside derivatives, *e.g.* diguanosinyl ethane. It is concluded that the outcome of exposure to such an agent depends upon the nature of the DNA modifications which it produces and of their susceptibility to repair.

Dr B. Singer (Berkeley) listed some of the chemically stable DNA–carcinogen adducts, and discussed their removal from DNA *in vivo* by enzymic mechanisms, noting that those adducts which persist during DNA replication have in many cases been shown to be mutagenic. In model studies of transcription using polynucleotides containing small numbers of modified bases, it has been found that 3-alkylcytosine, 3-alkylthymidine, 1-alkyladenine and all the $O$-alkylpyrimidines are transcribed but do not show base specificity, thus exhibiting partial or total ambiguity. This may arise from stacking interactions that can direct a base into the transcript without hydrogen bonding.

Dr R. Saffhill (Manchester) described *in vitro* systems for DNA synthesis in which both $O^4$-methylthymine and $O^6$-methylguanine miscode, leading to errors in the newly synthesized DNA. Other DNA methylation products, in particular 3-methylcytosine, $O^2$-methylthymine and 3-methylthymine, do not lead to a detectable number of errors. The differences obtained for these compounds in RNA- and DNA-synthesizing assays may reflect differences in the selectivity of the polymerases used. During DNA synthesis, miscoding by $O^6$-methylguanine is competitive with normal incorporation of cytosine and therefore sizes of DNA-precursor pools may be an important factor in carcinogenesis. When $O^4$-methylthymidine is present in cell cultures the incorporation of very small amounts of this modified nucleoside could be demonstrated, but when administered to rats $O^4$-methylthymidine (like 3-methylthymidine and $O^2$-methylthymidine) is excreted rapidly in an unchanged form.

During the afternoon of the second day consideration was given to the nature of the various repair processes *in vivo*. Professor W. G. Verly (Liege) discussed the repair of DNA containing apurinic sites on incubation with endonuclease VI, DNA polymerase I and ligase. Mammalian cells contain several apurinic endonuclease activities which may be successive maturation steps in the formation of the chromatin enzyme, the only one active on nuclear DNA. Repair deficiencies, eventually leading to high cancer susceptibility, might depend upon incomplete processing of these precursors. Proteins prepared from rat liver nuclei catalyse the disappearance of $O^6$-ethylguanine from DNA alkylated with ethylnitrosourea. Treatment of a copolymer of deoxythymidylic acid and $O^6$-methyldeoxyguanylic acid with these same nuclear proteins revealed that this repair was not due to release of free $O^6$-methylguanine, but to a more complex process involving the formation of a residue that remained in the polynucleotide and the release of an unidentified molecule.

Professor T. Lindahl (Gothenberg) gave several examples of the specificity of DNA-repair enzymes and described their properties in some detail. DNA glycosylases have been characterized that act upon DNA and release either 3-methyladenine or the imidazole-ring opened form of 7-methylguanine. *E. coli* mutants, deficient in the enzyme that releases 3-methyladenine (*tag* mutants), are sensitive to the action of methyl methanesulphonate and methylnitronitrosoguanidine, and have lost their ability to excise 3-methyladenine *in vivo*. $O^6$-Methylguanine is not removed by a DNA glycosylase but is altered to another, possibly non-coding, form *in situ* by an inducible *E. coli* enzyme.

Dr H. W. Thielman (Heidelberg) has developed a novel system which utilizes ether-permeabilized *E. coli* cells. With this system the induction of repair by carcinogens and mutagens has been assessed, and the enzymic activities involved in the excision and repair of carcinogen-induced DNA damage have been examined. Several normal and *xeroderma pigmentosum* fibroblast cell lines have been compared to one another after exposure to methyl methanesulphonate. The colony-forming ability and the capacity to introduce chain breaks into methylated DNA, but not apurinic DNA, were diminished in the *xeroderma pigmentosum* cells. This may result from lowered 3-methyladenine DNA glycosylase activity.

Professor A. E. Pegg (Hershey) gave a detailed appraisal of the enzyme responsible for removal of $O^6$-alkylguanine from liver DNA in rodents and humans. This is a unique enzyme which does not catalyse the removal of any other alkylated purines. Studies *in vivo* over a wide range of doses of the carcinogen dimethylnitrosamine have shown that the activity is progressively inhibited by the higher doses. On the other hand, it is stimulated by chronic exposure to low doses.

Activity is enhanced by growth hormone and thyroxine and is inhibited by $O^6$-alkylguanines. All of these properties, which were observed *in vivo* by following the loss of labelled $O^6$-methylguanine from DNA after treatment with dimethylnitrosamine, were also seen when the liver enzyme was assayed *in vitro*. There is evidence that this enzyme preparation has a limited capacity to catalyse this reaction, due to the lack of regeneration of an essential component.

The meeting ended with a wide-ranging discussion which included a survey of known and hypothetical lesions in DNA and their relevance to the initiation of carcinogenesis.

From the meeting overall, it appears that very detailed information on the role of DNA repair in chemical carcinogenesis has been obtained by studies with the *N*-nitroso compounds and related alkylating agents. From the analytical data, virtually all the reactions with DNA have been identified, and the importance of alkylation of *O*-atoms is now recognized. The potential of specific lesions to miscode has been established, using synthetic polynucleotides *in vitro*. Tissue differences in the repair of certain DNA adducts have been studied, and the specificities of these repair enzymes recognized, together with information on their induction and inhibition. Studies have been made on selected compounds from the other important classes of chemical carcinogens, polycyclic hydrocarbons and derivatives of aromatic amines. Several sites of reaction with DNA have been identified, though the analysis is not yet complete. There is evidence of long-term persistence of specific carcinogen–DNA adducts, and tissue differences in the repair of these lesions are under investigation. Progress has been made in improving the chromatographic procedures. However, the preparation of high-affinity antibodies for $O^6$-ethyldeoxyguanosine, allowing the detection of sub-picomole quantities suggests that radioimmunoassay may provide a rapid and much more sensitive technique for the detection of specific carcinogen–DNA adducts.

Generous support from the Christie Hospital Endowment Fund and the Cancer Research Campaign for the expenses of the Symposium is gratefully acknowledged.

PARTICIPANTS

| | | |
|---|---|---|
| P. J. Abbott | E. Kriek | M. F. Rajewsky |
| P. Brookes | L. G. Lajtha | R. Saffhill |
| M. M. Coombs | J. Laval | B. Singer |
| V. M. Craddock | T. Lindahl | B. W. Stewart |
| A. W. Craig | L. A. Loeb | A. Stone (observer) |
| L. Den Engelse | D. B. Ludlum | P. F. Swann |
| M. Fox | G. P. Margison | H. W. Thielmann |
| R. C. Garner | R. Montesano | W. G. Verly |
| A. Kinsella | P. J. O'Connor | T. Vickers (observer) |
| P. Kleihues | A. E. Pegg | |